# Psychometric evaluation of the 5-item Medication Adherence Report Scale questionnaire in persons with multiple sclerosis

**DOI:** 10.1371/journal.pone.0294116

**Published:** 2024-03-04

**Authors:** Maj Jožef, Igor Locatelli, Gregor Brecl Jakob, Lina Savšek, Katarina Šurlan Popovič, Žiga Špiclin, Uroš Rot, Mitja Kos

**Affiliations:** 1 Chair of Social Pharmacy, Faculty of Pharmacy, University of Ljubljana, Ljubljana, Slovenia; 2 Multiple Sclerosis Centre, Department of Neurology, University Medical Centre Ljubljana, Ljubljana, Slovenia; 3 Department of Neurology, General Hospital Celje, Celje, Slovenia; 4 Institute of Radiology, University Medical Centre Ljubljana, Ljubljana, Slovenia; 5 Chair of Neurology, Faculty of Medicine, University of Ljubljana, Ljubljana, Slovenia; 6 Laboratory for Imaging Technologies, Faculty of Electrical Engineering, University of Ljubljana, Ljubljana, Slovenia; University of Rijeka Faculty of Medicine: Sveuciliste u Rijeci Medicinski fakultet, CROATIA

## Abstract

The 5-item Medication Adherence Report Scale (MARS-5) is a reliable and valid questionnaire for evaluating adherence in patients with asthma, hypertension, and diabetes. Validity has not been determined in multiple sclerosis (MS). We aimed to establish criterion validity and reliability of the MARS-5 in persons with MS (PwMS). Our prospective study included PwMS on dimethyl fumarate (DMF). PwMS self-completed the MARS-5 on the same day before baseline and follow-up brain magnetic resonance imaging (MRI) 3 and 9 months after treatment initiation and were graded as highly and medium adherent upon the 24-cut-off score, established by receiver operator curve analysis. Health outcomes were represented by relapse occurrence from the 1^st^ DMF dispense till follow-up brain MRI and radiological progression (new T2 MRI lesions and quantitative analysis) between baseline and follow-up MRI. Criterion validity was established by association with the Proportion of Days Covered (PDC), new T2 MRI lesions, and Beliefs in Medicines questionnaire (BMQ). The reliability evaluation included internal consistency and the test-retest method. We included 40 PwMS (age 37.6 ± 9.9 years, 75% women), 34 were treatment-naive. No relapses were seen during the follow-up period but quantitative MRI analysis showed new T2 lesions in 6 PwMS. The mean (SD) MARS-5 score was 23.1 (2.5), with 24 PwMS graded as highly adherent. The higher MARS-5 score was associated with higher PDC (b = 0.027, *P<*0.001, 95% CI: (0.0134–0.0403)) and lower medication concerns (b = -1.25, *P<*0.001, 95% CI: (-1.93-(-0,579)). Lower adherence was associated with increased number (*P* = 0.00148) and total volume of new T2 MRI lesions (*P* = 0.00149). The questionnaire showed acceptable internal consistency (Cronbach α = 0.72) and moderate test-retest reliability (*r* = 0.62, *P* < 0.0001, 95% CI: 0.33–0.79). The MARS-5 was found to be valid and reliable for estimating medication adherence and predicting medication concerns in persons with MS.

## Introduction

Multiple sclerosis (MS) is a chronic, inflammatory, and demyelinating disease of the central nervous system that primarily affects the younger population in the 20–40 age range. It is around three times more prevalent in women compared to men in this age group [1]. The most common type of MS is relapsing-remitting (RRMS) in 85% of PwMS.

Oral disease-modifying therapies (DMTs) in MS offer a simple dosage form, increased efficacy, and safety. Dimethyl fumarate (DMF) is the most frequently prescribed oral DMT medication in Slovenia [2]. Its efficacy and safety have been confirmed in two multicentre randomized double-blind clinical trials DEFINE, CONFIRM, and follow-up trial ENDORSE [3–5].

MS requires continuous treatment monitoring. Brain magnetic resonance imaging (MRI) is the most sensitive surrogate marker of disease activity with its ability to detect clinically silent progression up to several years prior to clinical manifestation [6]. Quantitative MRI techniques enable objective brain MRI activity analysis and higher sensitivity. Unfortunately, they are not frequently used in routine clinical settings, due to complex validation procedures on different MR scanners [7].

Medication adherence (MA) is an essential part of MS treatment since nonadherence is associated with unfavourable health and economic outcomes [8]. There is no golden standard available. Therefore, a multimodal approach using different patient-reported outcomes (PRO) and MA measures is recommended [9]. MA estimation methods are divided into direct and indirect. Direct methods, such as drug concentrations in blood and biological markers are objective, but quite expensive and burdensome for patients. Commonly used indirect methods include questionnaires and MA measures estimated from medication dispenses. The Proportion of Days Covered (PDC) is the most accurate measure based on treatment gaps, defined as a ratio of adherent days to the total number of treatment days within the observation window. The main disadvantage is a complicated method of calculation [10]. Recently, a multimodal approach using different patient-reported outcomes (PRO) and MA measures has been recommended. Novel PRO tools are available in PwMS including a variety of Electronic Monitoring Devices (EMD) that could be used simultaneously as MA measures and interventions for improving MA [9].

Questionnaires are popular and inexpensive research methods for self-reported medication adherence assessment [11]. The Medication Adherence Report Scale-5 (MARS-5) is a simple and practical short version of the MARS-10 questionnaire that evaluates intentional and non-intentional causes of nonadherence [12]. It enables categorization, with higher scores indicating higher adherence. In the systematic review, Kwan et al. found moderate evidence for an accurate and reliable use of MARS-5 in health-care settlements [13]. The MARS-5 has been validated in patients with asthma, arterial hypertension, and diabetes [14].

The Beliefs about Medicines Questionnaire (BMQ) evaluates common beliefs about medicines. It is divided into two sections: (1) the general part assesses harms and overuse of medications (addiction and overprescribing), while (2) the specific part estimates beliefs towards treatment necessity. Both sections can be used in combination or independently from each other. The BMQ has been validated in patients with asthma, diabetes, and mental illnesses and evaluated in PwMS, as well [15, 16].

The psychometric evaluation of the questionnaire includes validity and reliability. Criterion validity refers to the level of association between the questionnaire scores and an independent criterion, for instance, clinical and patient-reported outcomes; thus, a valid adherence questionnaire can predict health outcomes based on the adherence score. Considering reliability, internal consistency is a measure of the inter-correlation between the statements of the questionnaire for a particular population, most commonly calculated as Cronbach α coefficient within the 0–1 range. Values at least 0.7 indicate acceptable internal consistency [17]. On the other hand, test-retest method is a measure of the temporal consistency that evaluates stability in time. It is calculated by intraclass correlation coefficient (Pearson *r*) if the same patients completed the same questionnaire twice at different time points. Values 0.75 and above indicate good reliability [18]. The period between tests should be optimal to avoid the memory effect, where patients respond by remembering the previous answer which can lead to overestimated reliability. To the best of our knowledge, there is no literature report regarding the validity of the MARS-5 questionnaire in PwMS. The primary aim of this study was to establish the criterion validity and reliability of the MARS-5 questionnaire in PwMS. We proposed the following hypothesis: (1) a lower score on MARS-5 is associated with lower weighted CMA7 and higher brain MRI activity, (2) a higher score on the MARS-5 is associated with stronger beliefs towards treatment necessity and (3) a lower score on the MARS-5 is associated with higher concerns regarding medication harm and overuse.

## Materials and methods

### Study design

**Fig 1:**
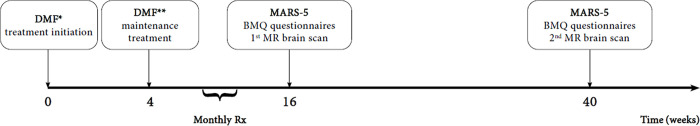
Timeline of the prospective study, *120 mg twice daily, **240 mg twice daily, Rx-refills.

We conducted a 1-year prospective cohort study that included PwMS from 3 Slovenian MS centres initializing DMF treatment from July 2021 to July 2022. At the DMF presentation interview, PwMS were invited to participate in a survey. Written consent was obtained from each patient. Demographical and clinical characteristics were collected from hospital medical charts. Baseline and follow-up brain MRIs were performed 3 and 9 months after the DMF treatment initiation. Health outcomes were evaluated as relapse occurrence after the beginning of DMF treatment and radiological progression between baseline and follow-up brain MRI based on radiologists’ examination and validated Quantim’s online brain MRI quantification service that includes new, enlarging, shrinking, and disappearing lesions, expressed as continuous variables (count and total volume) [19]. PwMS self-completed the MARS-5 and the BMQ questionnaires on the same day just before each brain MRI acquisition (Fig 1). We predicted the observation window long enough to avoid the memory effect. Medication-dispensing information was anonymously obtained from the National Outpatient Medicine Database based on patients’ date of birth. PwMS with identical dates of birth were identified by sex or date of the first DMF prescription. The dosage regimen of DMF in Slovenian PwMS is standardized with an initial dose of 120 mg twice daily. After 3 weeks, the maintenance dose of 240 mg twice daily is started.

Medication adherence was estimated as a Continuous multiple-interval medication availability (CMA7) measure by the AdhereR software package under an R environment with a predetermined gap [20]. The CMA7 is a PDC variant that allows carryover before and within the observation window [21]. If PwMS had several treatment episodes (i.e., exceeding the allowed gap), weighted CMA7 was used, based on episode duration. We assumed weighted CMA7 as an optimal choice for external validation with guaranteed adherence in the treatment episode. PwMS were graded as highly and medium adherent upon a predetermined threshold. Criterion validity of the MARS-5 questionnaire before follow-up brain MRI was established by association with weighted CMA7, radiological progression, and BMQ score as the main criterion attributes. We decided to evaluate the MARS-5 before the follow-up MRI due to the adequate length of the follow-up period. The reliability was assessed by internal and temporal consistency aiming at a specific MS population with predicted stable adherence over time.

### Study population

The study included male and female PwMS from three Slovenian Multiple Sclerosis Centres who were treatment-naive (initial DMT) or had switched from alternative DMTs from July 2021 to July 2022. Pregnant women were excluded. We proposed a moderate correlation (r = 0.5) between the MARS-5 score and the main criterion attribute; weighted mean CMA7. Statistical power analysis yielded to minimal cohort size of 38 PwMS (r = 0.5, β = 0.1, α (two-tailed) = 0.05).

### Variables and evaluation

The demographical characteristics were described by age and sex. Health outcomes after treatment initiation were evaluated qualitatively dichotomously by relapse occurrence within a 1-year follow-up period and active (new T2 and T1/Gadolinium (Gd) enhancing) lesions in the 6-month observation window, based on subjective radiologists’ examination that present the standard of care in routine clinical practice. The sensitive quantified radiological variables included the number and volume of the new and shrinking T2 MRI lesions in the 6-month observation window between baseline and follow-up brain MRI. The quantification of the baseline and follow-up brain MRI was evaluated synchronously followed by calculation of the variables by validated algorithms [19]. Other independent variables were treatment naivety, disease duration, and EDSS before DMF treatment initiation.

Medication adherence was assessed using a weighted CMA7 measure between baseline and follow-up MRI and MARS-5 score before the follow-up MRI. The treatment gap was set at 60 days and the adherence threshold at 90%. The observation window was defined from the date of the initial dispense till the date of the follow-up brain MRI. The MARS-5 questionnaire enables the evaluation of intentional and nonintentional medication-taking behaviour, described as forgetfulness, discontinuation, implementation, dosage, and general medication usage adjustment. Five claims are based on a 5-point Likert score (1 = always, 2 = often, 3 = sometimes, 4 = rarely, and 5 = never); therefore the total score lies within the 5–25 points range. A higher score signifies higher adherence. In the sensitivity analysis, the MARS-5 cut-off score was established based on association with the weighted CMA7 threshold [21].

Beliefs about medication treatment were assessed using the general and specific parts of the BMQ scale. The general part consists of two 4-item statements, while the specific part includes 11-item claims. Statements in both parts are based on the 5-point Likert score (1 = strongly disagree, 2 = disagree, 3 = uncertain, 4 = agree, 5 = strongly agree). Thus, the general score lies within the 8–40 points range, while the specific score is within the 11–55 points window. A higher score in the general part represents higher concerns regarding harm and overprescribing, while a higher score in the specific part represents stronger beliefs towards the treatment necessity of the prescribed medication [15]. Both questionnaires were translated into Slovenian language through forward/backward translation by two experts in the field of pharmacoepidemiology.

Criterion validity was evaluated by regression and correlation analysis between the MARS-5 score, BMQ score, and quantified radiological variables. The reliability was assessed by internal consistency and test-retest method over 6 months for the MARS-5 score at 3 and 9-month time points.

### Statistical analysis

Descriptive statistics for age, sex, treatment naivety, disease duration, and EDSS score before DMF treatment initiation were estimated. Counts and percentages were provided for categorical variables. Means and standard deviations were calculated for numerical variables. Power analysis with the assumed moderate association between MARS-5 score and weighted CMA7 was performed for minimal cohort size estimation.

Criterion validity was performed by employing a linear regression model for association with MARS-5 score, weighted CMA7 measure, and BMQ score, with MARS-5 score as a dependent variable. MARS-5 cut-off value was established by performing receiver operating characteristic (ROC) curve analysis based on the weighted CMA7 adherence thresholds. The relationship between medication adherence and radiological progression was evaluated by correlation analysis of the quantified radiological variables in highly and medium adherent groups using the Mann-Whitney U-test. The following covariates on the MARS-5 score were tested in each regression model: age, sex, treatment naivety, disease duration, and EDSS prior to treatment initiation. The final regression model was adjusted by stepwise method (back and forward adjusted) for optimal covariates selection. Internal consistency and test-retest evaluation were assessed by Cronbach α coefficient and intraclass correlation coefficient, for the MARS-5 score at baseline and follow-up brain MRI 3 and 9 months after treatment initiation. Statistical analysis was performed by R Studio version 2022.7.2.576 (R Studio Team 2022).

## Results

### Study population

Our study included 40 PwMS who met the inclusion criteria. Thirty-four PwMS were treatment-naive. The demographical and clinical characteristics are summarised in Table 1.

**Table 1:** PwMS demographic and clinical characteristics.

	PwMS
	N = 40
Female (%)	30 (75)
Mean age (SD)	37.6 (9.9)
Mean disease duration prior to DMF treatment (y, SD)	3.1 (4.8)
Treatment naive PwMS (%)	34 (85)
Mean EDSS prior to DMF treatment (SD)	1.04 (0.82)

### Health outcomes

None of the PwMS experienced a relapse during treatment in the 1-year follow-up period. Nine PwMS had radiological progression between the baseline and follow-up brain MR, based on radiologists’ examination. According to quantitative MRI analysis, 6 PwMS had new T2 lesions, with the mean new lesion volume (SD) of 0.128 (0.111) ml. Thirteen PwMS had shrinking lesions, with the mean decreased lesion volume difference (SD) of -0.0773 (0.0742) ml. The mean observation window duration (SD) between the baseline and follow-up brain MRI was 6.7 (1.2) months.

### Criterion validity

#### Medication adherence

**Fig 2:**
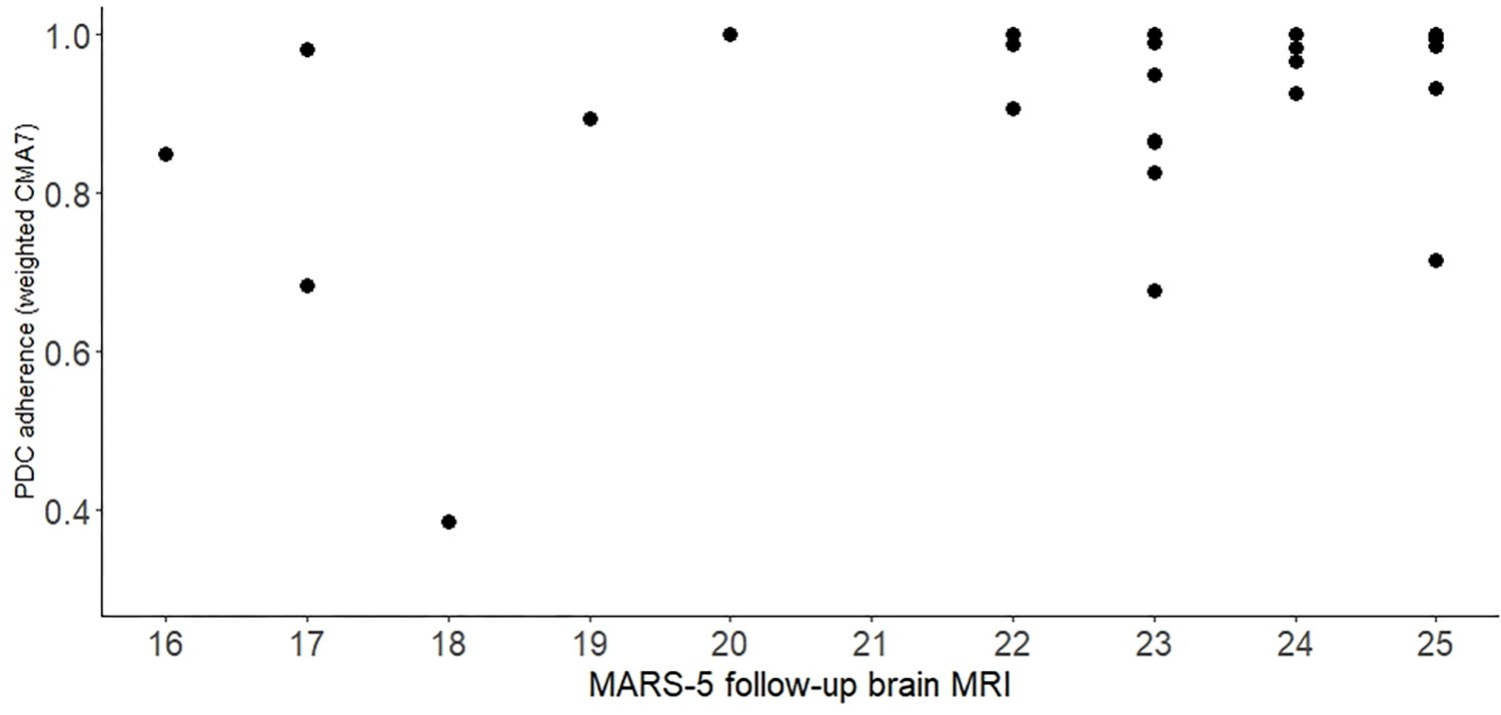
Scatterplot of the weighted CMA 7 measure and the MARS-5 at the follow-up brain MRI, number of PwMS N = 40.

The mean (SD) weighted CMA7 value during the 6-month observation window was 0.933 (0.125). Based on the 85 and 90% threshold, 34 and 31 PwMS were found to be highly adherent. The mean MARS-5 score (SD) before the follow-up MRI was 23.1 (2.5), respectively. The linear regression model showed a significant correlation between the MARS-5 score and weighted CMA7 measure (b = 0.027, *P*<0.001, 95% CI: 0.0134–0.0403). PwMS with a 1-point higher MARS-5 score was associated with a 2.7% higher weighted CMA7. The percentage of explained variance was 0.502. The correlation coefficient was 0.708. The scatterplot of MA is shown in Fig 2. The MARS-5 cut-off value was determined in subsequent ROC analysis based on the 85% and 90% weighted CMA7 threshold. At the weighted CMA7 ≥ 85%, maximized sensitivity, specificity, and positive predictive value (PPV) were attained at a MARS-5 score of ≥ 24 (sensitivity 67.6%, specificity 83.3%, PPV 80.2%). For the 90% threshold, sensitivity, and specificity were also optimized at the score of ≥ 24 (sensitivity 74.2%, specificity 88.9%, PPV 87.0%) and the ROC curve yielded an AUC value of 0.812 (*P* = 0.005, 95% CI 0.642–0.982). Therefore, a MARS-5 score of 24 was suggested as a cut-off score (Table 2). Based upon the cut-off score, 24 PwMS (60%) were found to be highly adherent with the mean MARS-score (SD) of 24.6 (0.5). The mean MARS-5 score (SD) in the lower adherence group was 20.6 (2.6). The association between age, sex, disease duration, and disability prior to DMF treatment initiation and MARS-5 score was explored by multiple logistic regression model. None of the covariates showed statistically significant odds for higher medication adherence based on the cut-off score.

**Table 2:** Comparison of the MARS-5 score and the weighted CMA7 adherence measure.

MARS score	PDC Adherence 85%*	PDC Adherence 90%*	MARS score	Mean PDC*
	TPR/FPR	TPR/FPR		
≥17	1.000/0.833	1.000/0.889	17	0.833
≥18	0.971/0.667	0.968/0.778	18	0.384
≥19	0.971/0.500	0.968/0.667	19	0.893
≥20	0.941/0.500	0.968/0.556	20	1
≥21	0.912/0.500	0.935/0.556	22	0.965
≥23	0.824/0.500	0.839/0.556	23	0.882
≥24†	0.676/0.167	0.742/0.111	24	0.981
= 25	0.412/0.167	0.452/0.111	25	0.975

TPR true positive rate (sensitivity), FPR false positive rate (1-specificity)

*weighted CMA 7

† cut-off value

#### Medication adherence in relation to health outcomes

Based on radiologists’ examination, a lower MARS-5 score was not associated with increased brain MRI activity (Mann-Whitney U-test, *P* = 0.077) between the baseline and follow-up brain MRI after treatment initiation. According to MRI quantification analysis, lower adherence (MARS-5 score < 24 points) was associated with an increased number (Mann-Whitney U-test, N = 40, *P* = 0.00148) and volume (Mann-Whitney U-test, N = 40, *P* = 0.00149) of the new brain MRI lesion between the baseline and follow-up brain MRI after DMF treatment initiation.

#### Medication adherence in relation to treatment beliefs

The mean score on BMQ (SD) was 19.5 (6.6) for the general and 34.1 (5.1) for the specific part, respectively. Correlation between the general and specific BMQ score and MARS-5 score was evaluated by linear regression model, with MARS-5 as a dependent variable. There was a statistically significant correlation between higher treatment concerns and nonadherence (b = -1.25, *P*<0.001, 95% CI: -1.93-(-0.579)). PwMS with a 1-point higher MARS-5 score had lowered the general BMQ score by 1.25 points. The percentage of explained variance was 0.509. The correlation coefficient was 0.713.

Stronger beliefs towards treatment necessity were not significantly related to higher adherence (b = -0.295, *P* = 0.369, 95% CI: -0.931–0.340). None of the covariates was significantly associated with higher concerns. The validity of the MARS-5 questionnaire is summarised in Table 3.

**Table 3:** Validity of the MARS-5 questionnaire.

	PwMS
	N = 40
MARS-5 mean Score (SD) at baseline	23.9 (1.64)
MARS-5 mean Score (SD) at follow-up	23.1 (2.49)
*Internal consistency*	0.72
Cronbach α	
*Reliability*	r = 0.62, *(0.33–0.79), *P*<0.0001,
Test-retest	
*Criterion validity*	
Weighted CMA7	†b = 0.027, *P<*0.001, *(0.0134–0.0403)
*Health outcome* **	
new T2 lesions (count)***	Mann-Whitney U-test, *P =* 0.00148
new T2 lesions (volume)***	Mann-Whitney U-test, *P =* 0.00149
*Patient-reported outcome*	
BMQ gen.	†b = -1.25, *P<*0.001, *(-1.93-(-0,579))
BMQ spec.	†b = -0.295, *P* = 0.369, * (-0.931–0.340)

† linear regression, b = slope coeff.

*95% CI

**6-month observation window

***MRI quantification

## Reliability evaluation

Internal consistency of the MARS-5 questionnaire was found to be acceptable (Cronbach α = 0.72) and test-retest evaluation resulted in a moderate intraclass correlated coefficient (*r* = 0.62, *P* < 0.0001, 95% CI: 0.33–0.79) at 3 and 9-month time points.

## Discussion

To the best of our knowledge, this is the first study establishing the psychometric properties of the MARS-5 questionnaire in PwMS. Early RRMS is frequently clinically silent between relapses. Consequently, there is a higher probability that PwMS start taking medication by their own judgement (reverse adherence). Therefore, we predicted the MARS-5 questionnaire to be suitable for PwMS because it enables nonintentional (1^st^ statement) and intentional (other 4 statements) nonadherence estimation. Criterion validity and reliability are essential for the routine implementation of the questionnaire in such a specific MS population with long-term DMTs over time.

Criterion validity was performed in terms of association with the MARS-5 score, weighted CMA7 measure, and new T2 MRI lesions (number and volume) between the baseline and follow-up brain MRI brain after treatment initiation, reaching the highest sensitivity possible. Criterion validity was confirmed by a statistically significant correlation of the MARS-5 score with the weighted CMA7 measure. The weighted CMA7 measure between the baseline and follow-up brain MRI was chosen because it includes carryover before and within the observation window and avoids medication dispenses inconsistency and falsely lowered adherence due to temporal adverse effects at the beginning of the treatment. Medication adherence using the MARS-5 score was evaluated at the follow-up brain MR due to the longer follow-up period and to avoid falsely higher values, since adherence declines after 6 months of treatment [22]. None of the demographical or clinical covariates was associated with medication adherence. This can be explained by the short follow-up period and small cohort size, which could lead to statistical non-significance. This is similar to the literature data where the correlation between age, sex, and adherence is generally inconclusive [23–25], although some studies report that older age is a strong predictor of adherence with males being more adherent than females, which is important since MS is more prevalent among women [22, 26, 27]. The MARS-5 score distribution was not normal but was generally above 20 points, similar to patients with ischemic bowel disease (IBD) [28]. The MARS-5 cut-off score was based on the association with weighted CMA7 85 and 90% threshold, respectively. The optimal sensitivity and specificity based on the 90% weighted CMA7 threshold were reached at MARS-5 scores of ≥ 24 points; thus 24 was suggested as a cut-off score. Our results are comparable to literature reports using the MARS-5 questionnaire in the treatment of IBD and secondary preventive treatment using statins in ischemic stroke [28, 29]. We find this comparison reasonable since both diseases also pose higher risks of intentional and nonintentional nonadherence.

Appropriate MS control follows the No Evidence Of Disease Activity (NEDA) paradigm [30]. Nine PwMS had radiological progression examined by radiologists between the baseline and follow-up brain MRI in a 6-month observation window after treatment initiation. Lower medication adherence was not related to a higher risk for radiological progression. This is probably due to the small cohort size and very short brain MRI observation window with a lower incidence of brain MRI activity which led to statistical nonsignificance. Namely, the results of our retrospective cohort study that included 164 participants where lower CMA7 for DMF were significantly associated with increased odds for radiological progression in a 12-month observation window based on radiologists’ examination [31]. According to quantitative MRI evaluation, 6 PwMS had new T2 lesions in the observation window and 5 PwMS had increased new lesion volume difference, exceeding the limit of quantification (0.03 ml), which can reliably differ between nonspecific and demyelinating lesions [19]. On the other hand, thirteen PwMS also had shrinking MRI lesions, which indirectly confirms DMF efficacy. Comparing both methods, a higher incidence of the brain MRI activity using routine clinical qualitative method is probably due to additional T1 active lesions which are not included in the quantitative MRI analysis protocol. Lower medication adherence was related to a higher risk for radiological progression, using validated quantitative variables. This can be explained by the higher sensitivity of the objective MRI quantitative techniques in a small sample population with a shorter observation window and adherence ceiling effect with a MARS-5 score distribution above 20 points. Therefore, we find the quantitative radiological variables as a more suitable measure for criterion validity compared to radiologists’ subjective impression. Unfortunately, quantitative MRI evaluation is currently not used routinely in clinical settings due to the adverse impact of MR scanner variability on the quantification of lesion volume and count differences, especially in cases where baseline and follow-up MR scanners are of different vendors or using different acquisition protocols [32].

The association between the MARS-5 score and the BMQ questionnaire was evaluated by linear regression. Our results showed that lower medication adherence was related to stronger concerns regarding medication harm and overprescribing. Higher adherence was not statistically significantly associated with stronger beliefs towards the treatment necessity of the prescribed medications. This is an expected finding since early MS is known to be asymptomatic between relapses, thus PwMS do not have a perception of treatment necessity. This is similar to literature data in other studies on MA and BMQ among PwMS [16, 33]. Our results are not consistent with previous studies that evaluated the necessity of taking medicines in stroke and asthma patients [34, 35]. One possible explanation is a different evolution of the symptoms in stroke survivors and patients with asthma exacerbation because of instant disability (i.e., hemiparesis and dyspnea).

Our study showed a high mean MARS-5 score exceeding 23 points at baseline and follow-up brain MRI. This confirms that self-reported adherence in our cohort is skewed toward perfect adherence. The reliability evaluation showed an acceptable Cronbach α coefficient (0.72). This is similar and comparable to the internal consistency measure in the original MARS-10 scale in patients with asthma (α = 0.65) and the MARS-5 scale in patients with arterial hypertension (α = 0.68) [14, 36]. Our result confirms the MARS-5 questionnaire as a reliable tool for the specific MS population with intentional and nonintentional adherence barriers on long-term DMTs. Regarding temporal consistency, the test-retest evaluation showed a moderate intraclass correlation coefficient at 2-time points, 6 months apart which can be explained by the asymptomatic initial phase of MS with lower radiological burden, when PwMS lose the perception of treatment necessity that outweighs the negative adverse effects experience after treatment initiation, as assumed by relative lower mean MARS-5 score at 9-month compared to 3-month time point. PwMS can start taking DMF by their own judgement due to delayed efficacy onset and twice daily dosage regimen. This is consistent with literature data that considers a significant decline in adherence 6 months after treatment initiation [22]. Adherence could have been affected by baseline MRI results, although we consider 6 months long enough to avoid the memory effect bias. Moderate test-retest reliability can also be explained by inherent within-person variability since MA is known to be a dynamic behavioural process due to person-time interaction and measurement error (i.e., some people take medications more frequently on particular days or months). This is similar to the studies regarding the variability of MA in time in patients with other chronic diseases treated with multiple medications [37, 38]. Our value is similar to the test-retest values using the original MARS-10 scale in patients with asthma (Pearson *r* = 0.65, 3 months apart) and MARS-5 scale in patients with IBD (Pearson *r* = 0.57, 1 year apart) [28, 36]. This is in contrast to Chan et al [14] reporting higher value in patients with arterial hypertension (Pearson *r* = 0.97, 2 weeks apart) which can probably be attributed to the memory effect. On the other hand, the results from the PHARMA-cop trial have shown temporal inconsistency at 2 measures over 1 year (Spearman test ρ = 0.103, *P* = 0.011) using the MARS-5 questionnaire in patients with chronic obstructive pulmonary disease, despite higher acceptable internal consistency (α = 0.77) and mean (SD) MARS-5 score of 23.49 (2.6) [39]. This result emphasizes the importance of temporal reliability evaluation in specific diseases with skewed self-reported adherence toward higher values. Considering challenging safety issues, the potential lack of medication-taking necessity perception in 6 months, and reports from other studies, we find the correlation strong enough to confirm the MARS-5 questionnaire as a moderately stable tool for assessing adherence in PwMS over time.

Our results should be viewed in light of the advantages and limitations of patient-reported outcomes. The strength of our study is the prospective design. PwMS self-completed the questionnaire on the same day just before each brain MRI; thereby avoiding potential bias due to the MRI results. The MARS-5 questionnaire is practical in clinical settings due to its short length. Instructions that precede the questions are structured in a way of reducing social desirability bias. Moreover, the Likert 5-scale score evaluation enables a broader nonadherence estimation compared to dichotomous yes/no answers.

We also have to consider some limitations of our study. Social desirability bias due to forgetfulness and memory effect could still have been present by the completion of both questionnaires. The observation window for brain MRI activity detection is very short. Routine MRI evaluation in clinical practice still relies on qualitative data, based on radiologists’ impressions. Quantitative techniques present the future of MRI disease monitoring in the short observation window, with standardization as a major drawback. Medication adherence may decline significantly after 6 months of treatment [22], thus a longer follow-up period in clinical settings is required. Additionally, the psychometric properties of the MARS-5 need to be evaluated in larger PwMS populations and other DMT medications, as well.

## Conclusions

The MARS-5 questionnaire was found to be reliable and valid for medication adherence assessment and predicting medication risks in PwMS in clinical settings. Criterion validity of the MARS-5 questionnaire was confirmed by association with the weighted CMA7 measure, radiological progression, and general part of the BMQ. Reliability was established by acceptable internal and moderate test-retest reliability. Results of the study in addition demonstrated high medication adherence in Slovenian PwMS treated with DMF.

## Supporting information

S1 Checklist STROBE 2007 (v4) statement—checklist of items that should be included in reports of *cohort studies*. (DOCX)

S1 Data (XLSX)

S2 Data (XLSX)

S3 Data (XLSX)

S1 File (R)
